# Mediterranean Diet Adherence and Genetic Background Roles within a Web-Based Nutritional Intervention: The Food4Me Study

**DOI:** 10.3390/nu9101107

**Published:** 2017-10-11

**Authors:** Rodrigo San-Cristobal, Santiago Navas-Carretero, Katherine M. Livingstone, Carlos Celis-Morales, Anna L. Macready, Rosalind Fallaize, Clare B. O’Donovan, Christina P. Lambrinou, George Moschonis, Cyril F. M. Marsaux, Yannis Manios, Miroslaw Jarosz, Hannelore Daniel, Eileen R. Gibney, Lorraine Brennan, Christian A. Drevon, Thomas E. Gundersen, Mike Gibney, Wim H. M. Saris, Julie A. Lovegrove, Keith Grimaldi, Laurence D. Parnell, Jildau Bouwman, Ben Van Ommen, John C. Mathers, J. Alfredo Martinez

**Affiliations:** 1 Department of Nutrition, and Food Science Physiology, Centre for Nutrition Research, University of Navarra, 31008 Pamplona, Spain; rsan.1@alumni.unav.es (R.S.-C.); snavas@unav.es (S.N.-C.); 2 CIBER Fisiopatología Obesidad y Nutrición (CIBERobn), Instituto de Salud Carlos III, 28023 Madrid, Spain; 3 Human Nutrition Research Centre, Institute of Cellular Medicine, Newcastle University, Newcastle upon Tyne NE1 7RU, UK; k.livingstone@deakin.edu.au (K.M.L.); carlos.celis@glasgow.ac.uk (C.C.-M.); john.mathers@newcastle.ac.uk (J.C.M.); 4 Hugh Sinclair Unit of Human Nutrition and Institute for Cardiovascular and Metabolic Research, University of Reading, Reading RG6 6AA, UK; a.l.macready@reading.ac.uk (A.L.M.); r.fallaize@reading.ac.uk (R.F.); j.a.lovegrove@reading.ac.uk (J.A.L.); 5 UCD Institute of Food and Health, UCD School of Agriculture and Food Science, University College Dublin, Belfield, 4 Dublin, Ireland; clare.odonovan@ucd.ie (C.B.O.); eileen.gibney@ucd.ie (E.R.G.); lorraine.brennan@ucd.ie (L.B.); mike.gibney@ucd.ie (M.G.); 6 Department of Nutrition and Dietetics, Harokopio University of Athens, 17671 Athens, Greece; cplambrinos@gmail.com (C.P.L.); gmoschi@hua.gr (G.M.); manios@hua.gr (Y.M.); 7 Department of Human Biology, NUTRIM School for Nutrition and Translational Research in Metabolism, Maastricht University Medical Centre, 6200MD Maastricht, The Netherlands; cyril.marsaux@gmail.com (C.F.M.M.); w.saris@maastrichtuniversity.nl (W.H.M.S.); 8 Department of Nutrition and Dietetic, Clinic of Metabolic Diseases and Gastroenterology, Institute of Food and Nutrition (IZZ), 02-903 Warsaw, Poland; jarosz@izz.waw.pl; 9 ZIEL Research Center of Nutrition and Food Sciences, Biochemistry Unit, Technische Universität München, 85354 Munich, Germany; hannelore.daniel@tum.de; 10 Department of Nutrition, Institute of Basic Medical Sciences, Faculty of Medicine, University of Oslo, 0317 Oslo, Norway; c.a.drevon@medisin.uio.no; 11 Vitas Analytical Services, Oslo Science Park, Gaustadalléen 21, 0349 Oslo, Norway; teg@vitas.no; 12 Eurogenetica Ltd., Burnham-on-Sea TA8 1HX, UK; keith.grimaldi@gmail.com; 13 Agricultural Research Service, USDA, Jean Mayer-USDA Human Nutrition Research Center on Aging at Tufts University, Boston, MA 02111, USA; Laurence.Parnell@ars.usda.gov; 14 TNO, Microbiology and Systems Biology, 3704HE Zeist, The Netherlands; jildau.bouwman@tno.nl (J.B.); ben.vanommen@tno.nl (B.V.O.); 15 Instituto de Investigaciones Sanitarias de Navarra (IdiSNA), 31008 Pamplona, Spain; 16 Instituto Madrileño de Estudios Avanzados (IMDEA) Alimentacion, 28049 Madrid, Spain

**Keywords:** Food4Me study, Mediterranean diet, genetic risk, obesity

## Abstract

Mediterranean Diet (MedDiet) adherence has been proven to produce numerous health benefits. In addition, nutrigenetic studies have explained some individual variations in the response to specific dietary patterns. The present research aimed to explore associations and potential interactions between MedDiet adherence and genetic background throughout the Food4Me web-based nutritional intervention. Dietary, anthropometrical and biochemical data from volunteers of the Food4Me study were collected at baseline and after 6 months. Several genetic variants related to metabolic risk features were also analysed. A Genetic Risk Score (GRS) was derived from risk alleles and a Mediterranean Diet Score (MDS), based on validated food intake data, was estimated. At baseline, there were no interactions between GRS and MDS categories for metabolic traits. Linear mixed model repeated measures analyses showed a significantly greater decrease in total cholesterol in participants with a low GRS after a 6-month period, compared to those with a high GRS. Meanwhile, a high baseline MDS was associated with greater decreases in Body Mass Index (BMI), waist circumference and glucose. There also was a significant interaction between GRS and the MedDiet after the follow-up period. Among subjects with a high GRS, those with a high MDS evidenced a highly significant reduction in total carotenoids, while among those with a low GRS, there was no difference associated with MDS levels. These results suggest that a higher MedDiet adherence induces beneficial effects on metabolic outcomes, which can be affected by the genetic background in some specific markers.

## 1. Introduction

An excessive Body Mass Index (BMI) is a well-established risk factor for metabolic diseases [1], including diabetes and cardiovascular diseases, and for other age-related diseases, including cancers and dementia [2,3,4,5]. Many studies have identified common triggers for unhealthy adiposity, such as changes in lifestyle, including higher consumption of saturated fats and sugar-rich foods, and reduced physical activity [6]. 

The investigation of dietary patterns, in relation to adiposity of a given population, may help to define nutritional factors affecting body fat deposition [7,8]. Indeed, both prospective and retrospective approaches concerning dietary pattern analysis have been used in different epidemiological studies [9,10]. In this context, the Mediterranean diet (MedDiet) is one of the most investigated dietary patterns, characterised by frequent consumption of vegetables and reduced amounts of animal products [11]. Large cohort studies involving MedDiet consumption have shown associations between diseases, such as obesity [12] or cardiovascular disease [13,14], and markers of homeostatic imbalance, like increased oxidative stress [15] or inflammatory status [16,17]. Recent findings have related a higher MedDiet adherence with a reduced incidence of metabolic syndrome (MetS) traits [18]. More specifically, MedDiet and some food components, such as olive oil and nuts, improve multiple metabolic biomarkers [11,19,20]—not only those related to body weight [21], but also to type-2 diabetes [22], hypertension [23], antioxidant status [24,25] and cancer [26].

In addition, the genetic background of a subject may have effects on their sensitivity for developing obesity [27]. Nutrigenetic studies identify those genetic markers that participate in the complex nutritional interactions that influence bodyweight and composition [28,29] and can form the basis of a personalized nutrition regimen. Interactions between the intake of specific nutrients and different single nucleotide polymorphisms (SNP) involved in diverse metabolic pathways have been identified [30]. However, genetic studies involving multiple trait disorders need further study in order to capture the multidimensional effects of genetic background in the clinical practice [31]. In this sense, several authors have tried to respond this need [32] by computing the genetic make-up through genetic risk scores (GRS) or genetic predisposition scores (GPS), based on summing the number of risk alleles [33]. These polygenetic scores may be useful for the evaluation of the risk in multiple diseases [34,35,36]. Indeed, a genome-wide association study highlighted the importance of several SNPs related to lipid metabolism in subjects with metabolic syndrome (MetS) [37]. The authors designed a GRS, strongly linked to MetS manifestations, but no pleiotropic effects were identified between lipid traits and other MetS components [37]. 

Previous investigations of interactions between GRS and dietary composition or habits have demonstrated that genotype may influence the impacts of saturated fat [38] and consumption of fried foods [39] or sugar-sweetened beverages [40] on measures of adiposity. However, investigations are needed to evaluate the efficacy of individualised diets with different macronutrient distributions and their interactions with the genetic background [41]. Based on this evidence, the aim of the present analysis was to explore the effects and interactions between MedDiet adherence and genetic background, by a multi-trait GRS involving relevant phenotypical and nutritional outcomes, throughout the Food4Me web-based nutritional intervention.

## 2. Materials and Methods

The present research is a secondary analysis from the Food4Me study [42], which included participants from 7 European countries [43]. The Food4Me study (http://www.food4me.org) was a European on-line randomized controlled intervention study, investigating the utility of a personalised nutrition approach for improving nutritional and diet-related outcomes, which has been described in detail elsewhere [42]. Briefly, the participants who met the inclusion criteria and completed the two-stage online screening, were randomised into the control group, or one of the 3 different intervention levels, which differed in personalization of dietary advice. General healthy eating information was provided to the participants included in the control group, while personalised nutritional advice was provided to the participants included in the 3 personalised nutrition advice groups, feedback was received based only on diet, diet and phenotype, or diet phenotype and genotype. These dietary recommendations were tailored for each individual, but were not specifically related to the MedDiet. 

For the present study, after statistical adjustments between the 4 intervention groups [43], the whole sample was merged together in order to assess the association between MedDiet adherence and changes in specific metabolic traits, and the influence of genetic load on these outcomes, between baseline and 6 months. 

**Figure 1:**
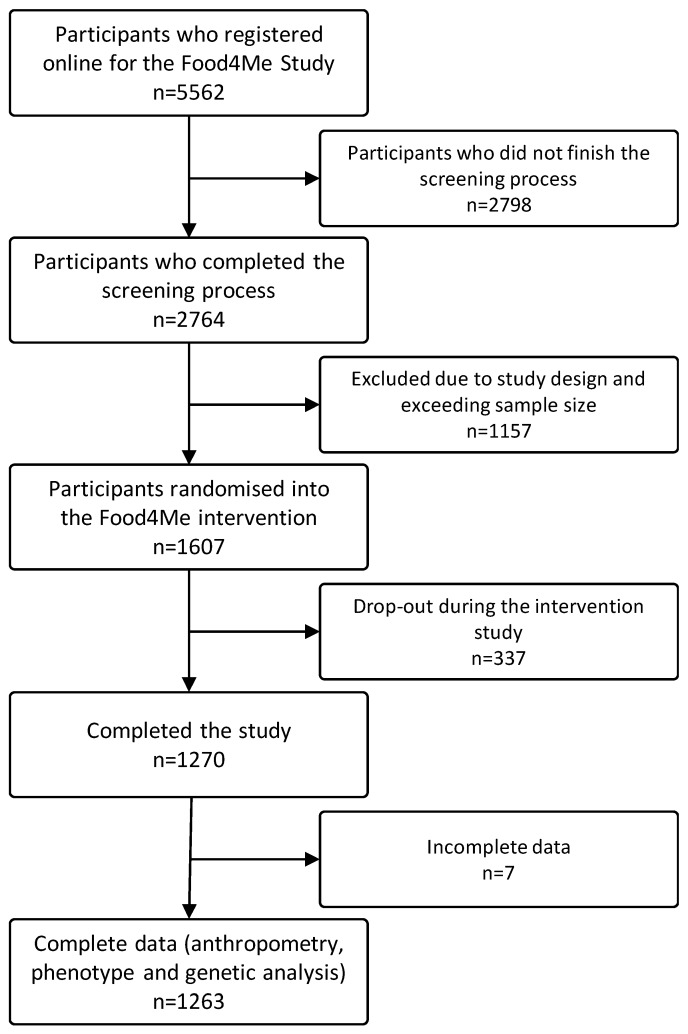
Flowchart of sample selection.

From the 1607 participants who were enrolled in the trial [42], 1270 completed the 6-month intervention [44]. All the volunteers who provided complete data on anthropometric measures, and provided adequate samples for biochemical and genetic analyses (*n* = 1263) were selected to be included in the current analyses (Figure 1).

A validated Food Frequency Questionnaire (FFQ) and dietary habits questionnaire [45,46,47] were completed by participants, to self-report usual dietary intake and dietary habits, at baseline and 6 months. Adherence to the MedDiet at baseline was calculated and used as an independent variable in all analyses carried out in the present study, except for the quantification of changes in MedDiet adherence and changes in metabolic traits, where data from 6 months were also used. Estimates of daily food intake were used to calculate Mediterranean Diet adherence by employing an adaptation of the PREDIMED fourteen-point score, previously described by Livingstone et al. [48], where the consumption or lack of intake of some foods related to the MedDiet pattern, were assessed. Similarly, self-reported anthropometrical measures were collected following validated procedures [49], at baseline and 6 months.

Blood samples were self-collected at baseline and 6 months by each participant, posted to the relevant recruitment centre and then shipped to a central analytical laboratory. Blood determinations were performed through the analysis of finger-prick samples, collected on Dried Blood Spots (DBS) cards, which were subsequently analysed by Vitas and DSM (Vitas Ltd., Oslo, Norway; DSM N.V., Heerlen, The Netherlands) for the determination of glucose, total cholesterol, carotenoids and fatty acid markers, as described [50,51]. For the present study, the biochemical measures were used to assess variations in metabolic traits related to obesity [52,53], as well as the assessment of variations in the adherence to the MedDiet [54]. Furthermore, buccal cell samples were collected at baseline by participants using buccal swabs. LGC Genomics (LGC, Teddington, UK) performed DNA isolation and the analyses of the samples by KASP^TM^ genotyping assays (LGC, Teddington, UK), following validated procedures.

All the participating centres obtained ethical approval for the study protocols from their corresponding local research ethics committees. The Food4Me project was registered with the Trial Registration number, NCT01530139, at clinicaltrials.gov (http://clinicaltrials.gov/show/NCT01530139). Furthermore, the signing of two online consent forms was required for all the candidates interested in participating in the Food4Me study.

A Genetic Risk Score (GRS) was computed “*a priori*” by adding the number of risk alleles presented for selected SNPs [55], which are related to metabolic syndrome traits. A preliminary selection process from the available SNPs in the Food4Me project was carried out to prevent spurious associations. The first stage was the determination of Hardy–Weinberg equilibrium through a Pearson chi-square test for each SNP (Table S1) using the *hwsnp* command [56]. We found the following SNPs: rs6323 for *MAOA* (*p*-value < 0.001), rs5082 for *APOA2* (*p*-value = 0.034), and rs708272 for *CETP* (*p*-value = 0.014) in disequilibrium, and so these three polymorphisms were not considered further. Subsequently, LDlink [57] (https://analysistools.nci.nih.gov/LDlink/) was used to determine Linkage Disequilibrium (LD) for those SNPs present in the same gene for the CEU population (Utah Residents from North and West Europe), in order to avoid collinearities in subsequent analyses. All SNPs were analysed for the following genes: *ADRB2* (rs1042713 and rs1042714); *AGT* (rs5051 and rs699); APOE (rs429358 and rs7412); *FTO* (rs1121980 and 9939609); *GC* (rs2282679, rs7041, and rs4588); and *CETP* (rs3764261 and 708272), which all presented *p*-values less than 0.005 for LD, except for *VDR* (rs1544410 and rs2228570), where the *p*-value was 0.984 for LD. For the SNPs within the same gene that were shown to be in high LD, those with the strongest association with relevant traits, based on published evidence (Genome-wide association studies -GWAS- or meta-analysis) were selected, i.e., rs1042714 [58], rs699 [59], rs7412 [60], rs9939609 [61,62,63], rs2282679 [64,65], and rs3764261 [60,66]. 

Finally, a Quantitative Trait Locus (QTL) analysis was performed on the remaining 21 SNPs, using the *qtlsnp* command [67], to assess the SNPs that presented associations with the metabolic traits included in the Food4Me study at baseline (BMI, waist circumference, glucose, total cholesterol, total carotenoids and Omega3 index) ,under a codominant assumption, and adjusted for age, gender, intervention centre, ethnicity, physical activity level, energy intake reported, smoking habits and occupation classification; random factors were selected based on previous literature evidence. From these analyses, a total of 14 SNPs with evidence of association with any relevant trait (Table S2) were selected for the final GRS composition.

Firstly, the estimated GRS and MDS at baseline were dichotomised at the medians to perform the subsequent analyses. Analysis of variance (ANOVA), adjusting by age and sex, as well as chi-square analyses, for continuous and categorical variables, respectively, were carried out to assess the differences in baseline characteristics between GRS and MDS levels. Supplementary linear regression mixed analyses were performed to investigate the association at baseline between anthropometric and biochemical markers with GRS and MDS as categorical variables, adjusting for confounders, including age, gender, centre of intervention, ethnicity, physical activity level, energy intake reported, smoking habits and occupation classification. Finally, in order to investigate changes, between baseline and month 6, in anthropometrical and biochemical measurements during the follow-up period, linear mixed models with repeated measurements were performed, also adjusting for confounders. In order to analyse the potential effects of interactions between MDS, GRS and time (baseline–6 months) on the outcome variables, the products of MDS × GRS × time were included in the linear mixed model. Statistical analyses were performed with STATA statistical software (Stata IC version 12.0, StataCorp., College Station, TX, USA), and *p*-values lower than 0.05 were considered statistically significant.

## 3. Results

### 3.1. Baseline Characteristics of the Sample and Associations of GRS and MDS

The GRS and MDS values were dichotomized at the medians to analyse differences in characteristics between higher or lower GRS and MDS at baseline (Table 1). A higher GRS was associated only with higher BMI. When the participants were categorised for baseline MDS, those with higher MDS values had significantly lower mean BMIs, waist circumference (WC) and total cholesterol, as well as greater physical activity, total carotenoid concentrations and Omega3 indices. 

The analysis of the interactions between GRS and MDS levels showed no significant interactions at baseline for BMI (*p* = 0.405), waist circumference (*p* = 0.973), physical activity factor (*p* = 0.470), energy intake (*p* = 0.412), glucose (*p* = 0.424), total cholesterol (*p* = 0.691), total carotenoids (*p* = 0.162) or Omega3 index (*p* = 0.394).

### 3.2. Associations of GRS and MDS at Baseline and after the Food4Me Intervention with Metabolic Traits

Effects of GRS, MDS and their interaction on metabolic traits were analysed for the changes in the Food4Me online intervention (Table 2). Total cholesterol was the only variable that showed differences depending on GRS levels during the follow-up period (low vs. high GRS: −0.21 ± 0.04 mmol/L vs. −0.09 ± 0.04 mmol/L; *p* = 0.043). On the other hand, significant differences were found for BMI (low vs. high MDS: −0.22 ± 0.04 kg/m^2^ vs. −0.40 ± 0.05 kg/m^2^; *p* = 0.011), WC (low vs. high MDS: −0.009 ± 0.002 m vs. −0.015 ± 0.002 m; *p* = 0.010) and glucose concentration (low vs. high MDS: −0.23 ± 0.04 mmol/L vs. −0.36 ± 0.04 mmol/L; *p* = 0.022), according to MDS levels of change during the 6 months, with a greater reduction for those participants who presented with higher MDS values. Moreover, total carotenoids showed significant interactions between GRS and MDS for the change from baseline.

**Figure 2:**
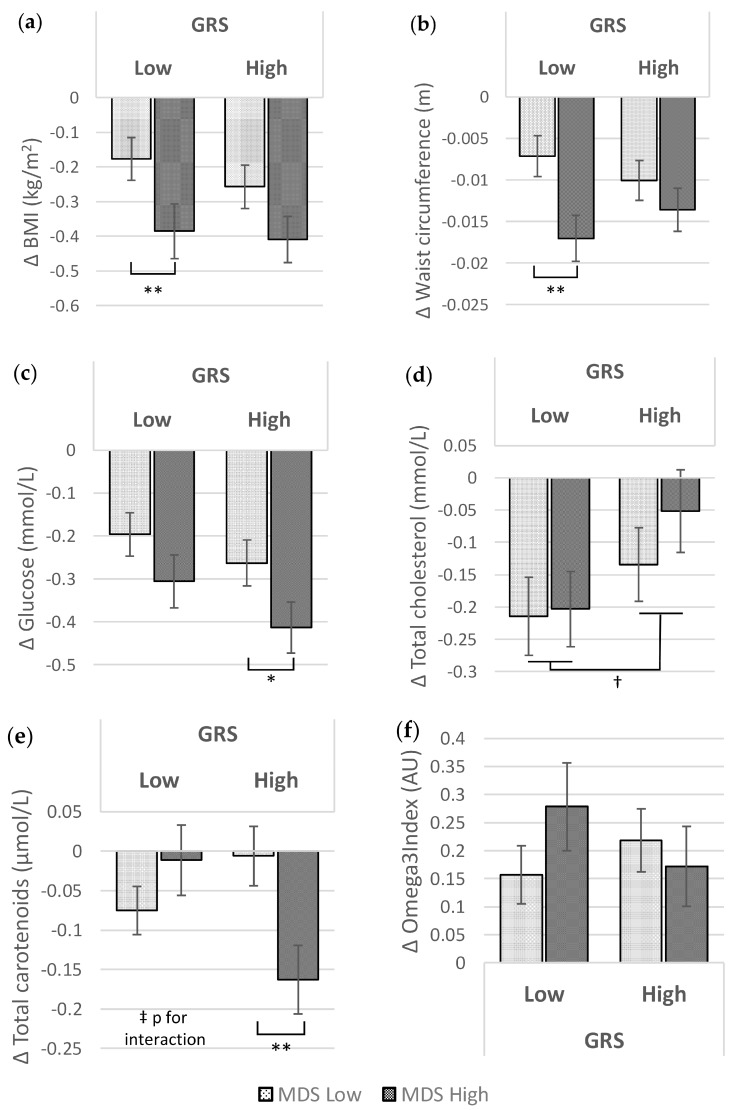
Differences in metabolic traits after the intervention (month 6 vs. baseline) between MDS levels, at each GRS level and differences between GRS levels, adjusted by age, gender, physical activity, country, ethnicity, smoking habit and energy intake reported: (**a**) BMI: *p*-value for interaction 0.688; (**b**) Waist circumference: *p*-value for interaction 0.226; (**c**) Glucose: *p*-value for interaction 0.718; (**d**) Total cholesterol: *p*-value for interaction 0.567; (**e**) Total carotenoids: *p*-value for interaction 0.006; (**f**) Omega3 index: *p*-value for interaction 0.209. *, ** and *** represent *p*-values less than 0.1, 0.05 and 0.001, respectively, for differences between MDS levels in each GRS level; and † represents *p*-values less than 0.05 for differences between GRS levels. ‡ *p*-value less than 0.05 for MDS × GRS interaction. MDS: Mediterranean Diet Score; GRS: Genetic Risk Score; AU: Arbitrary Units.

Subsequently, differences during the Food4Me intervention between the MDS at each level of the GRS were assessed (Figure 2). Greater results were detected in those volunteers with a low GRS when they presented a higher value in MDS for both anthropometrical measurements (Figure 2a: −0.176 kg/m^2^ vs. −0.385 kg/m^2^ with *p* = 0.048 for BMI; and Figure 2b: −0.007 m vs. −0.017 m with *p* = 0.010 for WC), but no interactions of GRS x MDS were detected (*p* for interaction = 0.689 for BMI and *p* for interaction = 0.226 for WC). However, the intervention caused slight differences for glucose in individuals with a high GRS, showing a greater reduction in those participants with a high MDS, although no significant differences were observed (Figure 2c: −0.306 mmol/L vs. −0.413 mmol/L with *p* = 0.061). Regarding cholesterol, a decrease after 6 months was observed (Figure 2d), being greater in those individuals with a lower GRS, independently of MDS level (*p*-value = 0.042).

Interestingly, concentrations of total circulating carotenoids showed significant differences depending on MDS level only for those volunteers with a high GRS. Those individuals with a high MDS exhibited a greater reduction than those with a low MDS in carotenoids during the follow-up period (Figure 2e: −0.006 μmol/L vs. −0.163 μmol/L, *p* = 0.007). No significant differences were detected for the Omega3 index (Figure 2f).

Sensitivity analyses (data not included) were performed, considering separately each SNP that was included in the GRS, to determine which specific SNPs might influence the interaction between GRS and MDS with total carotenoids in the blood. Significant differences were found between high and low MDS levels when participants carried both risk alleles for rs6564851 in *BCMO1* (−0.140; *p* = 0.0497), rs1042714 in *ADRB2* (−0.224; *p* = 0.016), and rs2228570 in *VDR* (−0.231; *p* = 0.042).

When MDS changes were analysed after the follow-up period in the Food4Me study, a global slight improvement in the overall score was observed (ΔMDS = 0.40 ± 0.05, *p* < 0.001), without differences based on the GRS levels. 

Finally, the associations between variations in MDS at 6 months were analysed, in order to determine overall changes in anthropometrical and biochemical variables as well as differences between subjects with low and high GRS (Table 3). Significant reductions were found for BMI (−0.07 ± 0.02 kg/m^2^), WC (−0.002 ± 0.001 m) and glucose concentrations (−0.05 ± 0.02 mmol/L) and no significant differences in changes were detected between the low and high levels of GRS for other variables.

## 4. Discussion

The present analysis adds value to previous results, associating genetic background disclosure with a higher adherence to MedDiet patterns, in a short-term intervention [68]. The current research evidenced the relevant influence of genetic background, assessed through a GRS, on obesity and some accompanying metabolic impairments (WC, cholesterol and glucose). Interestingly, this study demonstrated the beneficial effects of greater adherence to the MedDiet on anthropometric and biochemical markers, even in the presence of an elevated genetic risk.

Environmental factors, together with a higher GRS, have been found to interact with nutritional status [69]. Indeed, the baseline results suggest that an elevated GRS is associated with a higher body weight. These data agree with previous studies that found direct associations between genetic risk and body fatness indicators, as well as other related disorders [33,70]. As previously described by Vaxillaire et al. [71], polygenic-based scores for type 2 diabetes are associated with increased concentrations of fasting plasma glucose. Moreover, other authors have suggested that the increase in plasma glucose in individuals with elevated GRS and the susceptibility for developing type 2 diabetes could be associated with weight gain [72]. Our study suggests that MedDiet adherence at baseline generated beneficial effects on body weight during the Food4Me intervention, which may be partly associated with the reduction in blood glucose, regardless of GRS. This outcome agrees with recent results published by Ortega-Azorín et al. [73], who observed that individuals with a greater MedDiet adherence overcame the genetic risk on glucose levels, measured by the aggregated score of the risk alleles present in the *FTO* and *MC4R* genes. Additionally, the POUNDS LOST [74] and DPP [75] trials reported that individuals with a higher genetic risk for metabolic syndrome manifestations achieved greater benefits from a low-fat diet and an intensive lifestyle intervention based on weight reduction and increment of physical activity, respectively. 

These protective effects of the MedDiet on metabolic traits have also been described by other authors [21,76], and the interactions with specific SNPs have been tested for several traits, such as diabetes, blood pressure, lipid profiles or cardiovascular disease [13,77]. In this sense, the present study applied a GRS with genetically independent SNPs related to several features, such as body weight, adiposity, glucose homeostasis and lipid metabolism markers, assuming that multiple loci may jointly contribute to different traits in the presence of epistasis [78]. Similarly, a recent study tested the interactions between different GRS and a healthy diet score in a large cohort of 68,317 individuals of primarily European ancestry, concluding that the associations between genetic predisposition and obesity may be modulated by adherence to a healthy diet [79]. These findings are in accordance with the results obtained in our study, where the beneficial effects of the MedDiet during the intervention were more evident for anthropometrical measurements in those individuals with lower genetic loads. Interestingly, previous studies, focused on specific SNPs in *FTO* and *TCF7L2*, have reported a reduction in weight gain for participants who carried risk alleles, when they presented with high adherence to the MedDiet [70,80]. Furthermore, the study from Roswall et al. [70] found an association between risk alleles of *FTO* and BMI, although there were no changes during the follow-up period, which also agrees with the lack of association in anthropometrical changes during the follow-up period of our study.

Regarding biochemical outcomes, differences in GRS in our population were related to changes in the levels of total cholesterol, independently of MedDiet adherence. Indeed, all subjects reduced their cholesterol levels during the Food4Me intervention, although those individuals with a higher GRS presented a more discreet reduction than those with a low GRS, independently of MDS status. These outcomes may be partly explained by the metabolic dysregulation associated with the genetic background, which makes these individuals more susceptible to these responses, as has been seen in subjects with hypercholesterolemia treated with legumes [81]. In addition, our data agree with the results reported by Walker et al. [82], where the expected effect of body weight reduction on cholesterol concentration was attenuated by the genetic predisposition score for 36 SNPs related to lipid metabolism. Likewise in another study from the same authors, testing genetic predisposition scores, it was shown that higher concentrations of total cholesterol at baseline were associated with genetic predisposition, although no effects were found after an intervention period of 24 weeks with a diet reduced in saturated fatty acids [83].

Nevertheless, results on the influence of genetic load on nutritional status are still under debate [41]. Indeed, a recently published long-term study reported that a low fat diet modified the effects of a specific variant in *LIPC* on total cholesterol and other serum lipids [84]. Another analysis also found an interaction between *TCF7L2* and low adherence to the MedDiet, showing higher concentrations of total cholesterol for risk carriers [85]. These findings emphasise the need to design more studies that identify the interactions between macronutrient intake or dietary patterns and genetic make-up, with the main focus on facilitating clear and evidence-based knowledge to practitioners to allow an easier translation to clinical and population settings through personalised nutrition [41]. Moreover, the present results might suggest the presence of interactions between some alleles of various genes with roles in lipid metabolism, leading to a greater impact on phenotypes [86]. Further research on gene–gene interactions may provide better understanding of individual variation in response to different diets [87].

On the other hand, levels of carotenoids and Omega3 index at baseline were higher in volunteers with a higher MDS, supporting the increased consumption of fruit, vegetables [88] and oily fish [89] during the trial at baseline. Despite the positive initial association between MDS and total carotenoid concentration, a global carotenoid reduction and also an interaction of MDS and carotenoids after the Food4Me intervention was detected. Dietary carotenoids share absorptive pathways with dietary lipids [90], and concentrations in plasma have been positively associated with cholesterol concentration in blood and adiposity [91,92], which may suggest a parallelism with the results obtained concerning total cholesterol and body weight reduction observed in our study. Furthermore, associations between weight reduction and a decrease in carotenoids [93] and lycopene [94] levels have been found after weight loss interventions with caloric restriction for 4 weeks or 12 weeks. These reports help to explain the results observed in our study, which was not specifically designed for weight loss. Furthermore, interactions with effects on carotenoids have been described for *BCMO1* [95] and other genes related to lipid metabolism [90]. These results demonstrate that considering the genotype in personalised dietary advice may improve the effect on dietary interventions and may help to achieve an adequate status in nutritional biochemical markers [96]. Similarly, the influences of SNP interactions with low responses to plasma β-carotene uptake/conversion, even after administrating specific doses, have been described [97]. Interestingly, this research has identified that concentrations of carotenoids follow different trends in low GRS compared with individuals presenting with a high GRS. This interaction should be taken into consideration, because individuals with a high MDS also report lower carotenoid levels, which agree with lower levels in other lipid markers. The interplay between MDS and genetic make-up, based on risk allele analysis, may contribute to the development of more accurate personalised nutrition advice. 

A possible limitation of the present analysis is that individual deviations in dietary intake due to personalised advice, based on specific nutrients, could not be taken into account; thus, further prospective analyses in nutritional interventions designed for specific nutrient consumption may be needed. Another limitation is the incomplete nature of GRS, since other genetic variants with roles in the response to MDS remain to be described. In addition, it must also be noted that the Food4Me study was not designed as a weight-loss nutritional intervention. Thus, results observed on anthropometric changes should be considered with caution, although it cannot be forgotten that the project was focused on healthy eating guidance. Nevertheless, our study included sensitivity analyses by adjusting for the different intervention levels of advice in the Food4Me study, to evaluate the robustness of the results and no significant differences were found at any level. 

## 5. Conclusions

The results of the present study showed that baseline MedDiet adherence is associated with beneficial effects on anthropometrical measurements and might overcome an adverse genetic load. Nevertheless, a higher GRS may reduce these benefits on total cholesterol concentration, while the levels of carotenoids in plasma have been shown in the Food4Me population to be affected by the interplay of GRS and MedDiet adherence. Indeed, gene × nutrient interactions may contribute to the development of feasible precise nutrigenetic advice, by which the interactions of genetic make-up and the dietary features can be incorporated. 

## Figures and Tables

**Table 1:** Baseline characteristics of the volunteers selected from the Food4Me, and differences between categorized at median GRS and MDS levels (*low score* vs. *High score)*.

	Overall		GRS				*p* ^†^	MDS				*p* ^†^
			*Low*		*High*			*Low*		*High*		
*n* (female)	1263	(722)	640	(354)	623	(368)	0.177	747	(419)	516	(303)	0.353
Age (years)	40.8	±13.0	41.2	±12.8	40.4	±13.1	0.357	40.2	±12.9	41.7	±13.0	**0.034**
Ethnicity *n* (% Caucasians)	1224	(96.9%)	618	(96.6%)	606	(97.3%)	0.397	730	(97.7%)	494	(95.7%)	0.230
Smoke habit *n* (%)												
Never smoker	781	(61.8%)	392	(61.3%)	389	(62.4%)	0.902	473	(63.3%)	308	(59.7%)	0.294
Former smoker	333	(26.4%)	172	(26.9%)	161	(25.8%)		185	(24.8%)	148	(28.7%)	
Smoker	149	(11.8%)	76	(11.9%)	73	(11.7%)		89	(11.9%)	60	(11.6%)	
MDS (over 14)	5.1	±1.7	5.1	±1.6	5.2	±1.7	0.529	4.0	±1.0	6.8	±0.9	**<0.001**
GRS (over 28)	10.5	±2.3	8.6	±1.3	12.4	±1.3	**<0.001**	10.5	±2.3	10.5	±2.4	0.974
BMI (kg/m^2^)	25.4	±4.7	25.2	±4.5	25.6	±4.8	**0.018**	25.6	±4.7	25.1	±4.6	**0.012**
Waist circumference (m)	0.859	±0.136	0.857	±0.133	0.861	±0.140	0.052	0.866	±0.138	0.848	±0.134	**0.001**
Physical activity factor (AU)	1.521	±0.104	1.525	±0.106	1.517	±0.101	0.094	1.516	±0.104	1.527	±0.103	**0.021**
Energy intake reported (kcal/day)	2552	±1066	2609	±1086	2493	±1042	0.079	2512	±1060	2609	±1072	0.069
Glucose (mmol/L)	3.73	±0.80	3.69	±0.80	3.77	±0.79	0.067	3.71	±0.75	3.76	±0.86	0.499
Total cholesterol (mmol/L)	4.61	±0.95	4.61	±0.93	4.60	±0.97	0.601	4.64	±0.96	4.55	±0.93	**0.008**
Total carotenoids (μmol/L)	1.52	±0.67	1.50	±0.64	1.55	±0.71	0.285	1.45	±0.60	1.64	±0.76	**<0.001**
Omega3 index (AU)	5.71	±1.22	5.70	±1.20	5.73	±1.24	0.377	5.53	±1.08	5.97	±1.35	**<0.001**

Continuous values, expressed as mean ± standard deviation. † *p*-value for differences between levels of dichotomised GRS and MDS, chi square test was carried out for categorical variables and ANOVA, adjusting for age and sex for continuous variables. BMI: Body Mass Index. MDS: Mediterranean Diet Score. GRS: Genetic Risk Score. AU: Arbitrary Units.

**Table 2:** Anthropometrical and biochemical changes at 6 months into the Food4Me intervention, depending on GRS and MDS categories at baseline.

	***Baseline GRS Category***						***p*^‡^*for Differences***
	***Low***		***p*^†^**	***High***		***p*^†^**	
BMI (kg/m^2^)	−0.281	±0.047	**<0.001**	−0.333	±0.044	**<0.001**	0.417
Waist circumference (m)	−0.012	±0.002	**<0.001**	−0.012	±0.002	**<0.001**	0.920
Glucose (mmol/L)	−0.251	±0.039	**<0.001**	−0.338	±0.039	**<0.001**	0.114
Total cholesterol (mmol/L)	−0.209	±0.040	**<0.001**	−0.093	±0.041	**0.024**	**0.043**
Total carotenoids (μmol/L)	−0.043	±0.026	0.102	−0.085	±0.028	**0.003**	0.282
Omega3 index (AU)	0.217	±0.045	**<0.001**	0.195	±0.044	**<0.001**	0.718
	***Baseline MDS Category***						***p*^§^*for Differences***
	***Low***		***p*^†^**	***High***		***p*^†^**	
BMI (kg/m^2^)	−0.217	±0.044	**<0.001**	−0.397	±0.052	**<0.001**	**0.011**
Waist circumference (m)	−0.009	±0.002	**<0.001**	−0.015	±0.002	**<0.001**	**0.010**
Glucose (mmol/L)	−0.230	±0.036	**<0.001**	−0.360	±0.043	**<0.001**	**0.022**
Total cholesterol (mmol/L)	−0.174	±0.042	**<0.001**	−0.127	±0.044	**0.003**	0.453
Total carotenoids (μmol/L)	−0.041	±0.024	0.097	−0.087	±0.031	**0.005**	0.244
Omega3 index (AU)	0.187	±0.038	**<0.001**	0.225	±0.053	**<0.001**	0.573

† *p*-value for quantifying the effects of the intervention (time 0 vs time 6-months), adjusted by age, gender, physical activity, country, ethnicity, smoke habit and energy intake reported. ‡ *p*-value for differences between the effects of low and high level of GRS. § *p*-value for differences between the effects of low and high levels of MDS. GRS: Genetic Risk Score; MDS: Mediterranean Diet Score; BMI: Body Mass Index; AU: Arbitrary Units.

**Table 3:** Overall effect of MDS variation on anthropometrical and biochemical traits of the Food4Me volunteers during the intervention, and based on GRS categories.

			*p* ^†^	GRS				*p* ^‡^
	Overall			Low		High		
BMI (kg/m^2^)	−0.065	±0.022	**0.003**	−0.060	±0.024	−0.069	±0.021	0.457
Waist circumference (m)	−0.002	±0.001	**0.003**	−0.002	±0.001	−0.002	±0.001	0.709
Glucose (mmol/L)	−0.050	±0.017	**0.003**	−0.043	±0.018	−0.057	±0.017	0.182
Total cholesterol (mmol/L)	0.006	±0.018	0.753	−0.004	±0.018	0.015	±0.018	0.058
Total carotenoids (μmol/L)	−0.018	±0.012	0.118	−0.014	±0.012	−0.023	±0.012	0.206
Omega3 index (AU)	−0.010	±0.019	0.611	−0.007	±0.021	−0.012	±0.020	0.677

Values expressed as adjusted mean of change ± standard error for each point of variation of MDS. † *p*-value for effects of MDS variation during the 6-month period. ‡ *p*-value for differences in the changes between GRS levels. BMI: Body Mass Index. MDS: Mediterranean Diet Score. GRS: Genetic Risk Score. AU: Arbitrary Units.

## Supplementary Materials

The following are available online at www.mdpi.com/2072-6643/9/10/1107/s1, Table S1: SNP distribution and Hardy-Weinberg test. Table S2: QTL association analysis with multiple traits at baseline.
