# Supplementary material for: Mediterranean Diet Adherence and Genetic Background Roles within a Web-Based Nutritional Intervention: The Food4Me Study
Source: Nutrients. 2017 Oct 11;9(10):1107. doi: 10.3390/nu9101107 (PMC5691723; doi:10.3390/nu9101107)
Supplement: Supplementary file 1 [file nutrients-09-01107-s001.zip › nutrients-225762-supplementary.pdf]

# Supplementary Materials: Mediterranean diet adherence and genetic background roles within a web-based nutritional intervention: the Food4Me study

**Rodrigo San-Cristobal, Santiago Navas-Carretero, Katherine M Livingstone, Carlos Celis-Morales, Anna L Macready, Rosalind Fallaize, Clare B O'Donovan, Christina P. Lambrinou, George Moschonis, Cyril F.M. Marsaux, Yannis Manios, Miroslaw Jarosz, Hannelore Daniel, Eileen R Gibney, Lorraine Brennan, Christian A Drevon, Thomas E Gundersen, Mike Gibney, Wim HM Saris, Julie A Lovegrove, Keith Grimaldi, Laurence D. Parnell, Jildau Bouwman, Ben van Ommen, John C Mathers, J. Alfredo Martinez, on behalf of the Food4Me Study.**

## 2. Materials and Methods

**Table S1.** SNP distribution and Hardy-Weinberg test

| Gene | Polymorphism | Common alelle / Risk alelle | Heterozygous (%) | Homozygous for risk allele (%) | $X^2$ † | p † | Exact significance |
|---|---|---|---|---|---|---|---|
| *ADRB2* | rs1042713 | G / A | 49.33 | 15.6 | 0.818 | 0.3659 | 0.3807 |
| *ADRB2* | rs1042714 | C / G | 49.05 | 13.95 | 1.642 | 0.2001 | 0.2121 |
| *AGT* | rs5051 | C / T | 48.18 | 21 | 0.924 | 0.3365 | 0.3343 |
| *AGT* | rs699 | T / C | 47.57 | 21.16 | 1.884 | 0.1699 | 0.1716 |
| *APOA1* | rs5069 | C / T | 6.74 | 0.08 | 0.178 | 0.6727 | 1 |
| *APOA2* | rs5082 | T / C | 44.17 | 15.62 | 4.502 | **0.0339** | **0.0358** |
| *APOA5* | rs662799 | A / G | 13.88 | 0.79 | 0.944 | 0.3313 | 0.3214 |
| *APOE* | rs429358 | T / C | 24.54 | 2.06 | 0 | 0.9889 | 1 |
| *APOE* | rs7412 | T / C | 11.91 | 87.85 | 0.79 | 0.3742 | 0.6233 |
| *BCMO1* | rs6564851 | G / T | 49.64 | 28.55 | 0.009 | 0.9263 | 0.9549 |
| *CETP* | rs3764261 | G / T | 44.15 | 9.79 | 0.358 | 0.5496 | 0.6032 |
| *CETP* | rs708272 | C / T | 45.99 | 21.6 | 6.057 | **0.0138** | **0.0142** |
| *COMT* | rs4680 | G / A | 49.8 | 24.82 | 0.02 | 0.8887 | 0.9102 |
| *FADS1* | rs174546 | C / T | 46.43 | 10.54 | 1.848 | 0.174 | 0.1864 |
| *FTO* | rs9939609 | T / A | 50.04 | 18.05 | 0.525 | 0.4689 | 0.4913 |
| *FTO* | rs1121980 | C / T | 49.68 | 21.16 | 0 | 0.9981 | 1 |
| *GC* | rs2282679 | A / C | 38.44 | 7.58 | 0.512 | 0.4742 | 0.472 |
| *GC* | rs4588 | C / A | 40.86 | 7.92 | 0.042 | 0.838 | 0.8898 |
| *GC* | rs7041 | G / T | 48.81 | 18.94 | 0.048 | 0.8268 | 0.8189 |
| *GPX1* | rs1050450 | C / T | 42.71 | 10.36 | 0.243 | 0.6221 | 0.6482 |
| *GSTP1* | rs1695 | G / A | 43.93 | 43.3 | 1.212 | 0.2709 | 0.2643 |
| *IRS1* | rs1801278 | G / A | 13.4 | 0.56 | 0.023 | 0.88 | 0.8337 |
| *LIPC* | rs10468017 | C / T | 40.33 | 7.45 | 0.099 | 0.7526 | 0.7786 |
| *MAOA* | rs6323 | T / G | 25.54 | 18.4 | 206.679 | **<0.001** | **<0.001** |
| *MTHFR* | rs1801133 | C / T | 44.42 | 10.93 | 0.007 | 0.932 | 0.9495 |
| *SLC6A4* | rs16965628 | G / C | 13.73 | 0.48 | 0.107 | 0.7433 | 1 |

**Table S1.** *Cont.*

| | | | | | | | |
|---|---|---|---|---|---|---|---|
| *SOD2* | rs4880 | T / C | 50.75 | 25.34 | 0.295 | 0.5872 | 0.612 |
| *TCF7L2* | rs7903146 | C / T | 42.11 | 7.22 | 1.847 | 0.1741 | 0.1874 |
| *TPH2* | rs4570625 | G /T | 34.31 | 4.21 | 0.563 | 0.4529 | 0.5023 |
| *VDR* | rs1544410 | G / A | 48.33 | 16.67 | 0 | 0.9919 | 1 |
| *VDR* | rs2228570 | C / T | 47.27 | 14.89 | 0.005 | 0.9429 | 0.9526 |

† $X^2$ and p-value for Hardy-Weinberg equilibrium test

**Table S2.** QTL association analysis with multiple traits at baseline

| | **Additive effect** | | **Dominant effect** | | **Both†** | |
|---|---|---|---|---|---|---|
| | F | p | F | p | F | p |
| *BMI (kg/m²)* | | | | | | |
| rs9939609 | 5.85 | **0.016** | 0 | 0.961 | 3.02 | **0.049** |
| rs1801133 | 0.24 | 0.622 | 6.11 | **0.014** | 3.31 | **0.037** |
| rs4570625 | 4.76 | **0.029** | 2.53 | 0.112 | 2.38 | 0.093 |
| rs6564851 | 5.01 | **0.025** | 0.03 | 0.873 | 2.51 | 0.082 |
| *Waist circumference (m)* | | | | | | |
| rs9939609 | 5.3 | **0.021** | 0.98 | 0.323 | 2.79 | 0.062 |
| rs1801133 | 0.72 | 0.397 | 7.47 | **0.006** | 3.83 | **0.022** |
| rs4570625 | 6.78 | **0.009** | 2.31 | 0.129 | 3.51 | **0.03** |
| *Glucose (mmol/L)* | | | | | | |
| rs1042714 | 10.51 | **0.001** | 2.7 | 0.101 | 5.44 | **0.004** |
| rs16965628 | 4.15 | **0.042** | 3.99 | **0.046** | 2.12 | 0.121 |
| rs2282679 | 0.81 | 0.368 | 4.18 | **0.041** | 2.15 | 0.117 |
| rs4680 | 5.55 | **0.019** | 1.85 | 0.175 | 3.73 | **0.024** |
| *Total cholesterol (mmol/L)* | | | | | | |
| rs7903146 | 6.11 | **0.014** | 0.59 | 0.444 | 3.41 | **0.033** |
| rs7412 | 5.18 | **0.023** | 10.22 | **0.001** | 8.73 | **<0.001** |
| rs1050450 | 6.05 | **0.014** | 7.26 | **0.007** | 4.53 | **0.011** |
| *Total carotenoids (µmol/L)* | | | | | | |
| rs7412 | 1.18 | 0.278 | 0.05 | 0.823 | 4.3 | **0.014** |
| rs662799 | 4.95 | **0.026** | 2.03 | 0.154 | 3.22 | **0.04** |
| *Omega3 index (AU)* | | | | | | |
| rs2228570 | 5.86 | **0.016** | 0.01 | 0.928 | 3.17 | **0.042** |
| rs4880 | 0.68 | 0.411 | 5.52 | **0.019** | 3.17 | **0.042** |

† Analysis assuming that both effects are equal to zero, which is equivalent to comparing means across the three possible genotypes and phenotypes. AU: Arbitrary units. QTL: Quantitative Trait Locus.
